# Divergence in Endothelin-1- and Bradykinin-Activated Store-Operated Calcium Entry in Afferent Sensory Neurons

**DOI:** 10.1177/1759091415578714

**Published:** 2015-03-27

**Authors:** Kalina Szteyn, Ruben Gomez, Kelly A Berg, Nathaniel A Jeske

**Affiliations:** 1 Department of Oral and Maxillofacial Surgery, University of Texas Health Science Center at San Antonio, TX, USA; 2 Department of Pharmacology, University of Texas Health Science Center at San Antonio, TX, USA; 3 Department of Physiology, University of Texas Health Science Center at San Antonio, TX, USA

**Keywords:** bradykinin, endothelin, inflammation, pain, SOCE, trigeminal neurons

## Abstract

Endothelin-1 (ET-1) and bradykinin (BK) are endogenous peptides that signal through Gαq/11-protein coupled receptors (GPCRs) to produce nociceptor sensitization and pain. Both peptides activate phospholipase C to stimulate Ca^2+^ accumulation, diacylglycerol production, and protein kinase C activation and are rapidly desensitized via a G-protein receptor kinase 2-dependent mechanism. However, ET-1 produces a greater response and longer lasting nocifensive behavior than BK in multiple models, indicating a potentially divergent signaling mechanism in primary afferent sensory neurons. Using cultured sensory neurons, we demonstrate significant differences in both Ca^2+^ influx and Ca^2+^ release from intracellular stores following ET-1 and BK treatments. As intracellular store depletion may contribute to the regulation of other signaling cascades downstream of GPCRs, we concentrated our investigation on store-operated Ca^2+^ channels. Using pharmacological approaches, we identified transient receptor potential canonical channel 3 (TRPC3) as a dominant contributor to Ca^2+^ influx subsequent to ET-1 treatment. On the other hand, BK treatment stimulated Orai1 activation, with only minor input from TRPC3. Taken together, data presented here suggest that ET-1 signaling targets TRPC3, generating a prolonged Ca^2+^ signal that perpetuates nocifensive responses. In contrast, Orai1 dominates as the downstream target of BK receptor activation and results in transient intracellular Ca^2+^ increases and abridged nocifensive responses.

## Introduction

Endothelin-1 (ET-1) and bradykinin (BK) are two endogenous neuropeptides involved in inflammatory and cancer pain (Mantyh et al., 2002; Viet and Schmidt, 2012). ET-1 was first isolated and described as a powerful vasoconstrictor in 1988 (Masaki, 2004) and is synthesized by multiple cell types. ET-1 signals through two Gαq-protein coupled receptors (GPCRs), endothelin-A (ET_A_R), and endothelin-B (ET_B_R), which are expressed in a tissue-dependent manner (Stephenson et al., 1994; Takigawa et al., 1995; Webb, 1997; Khodorova et al., 2009). The affinities of the three isoforms of endothelin (ET-1, -2, and -3) also differ for ET_A_R and ET_B_R. ET_B_R binds all three endothelin isoforms with equal affinity, whereas ET-1 has high affinity for ET_A_R over ET_B_R (Khodorova et al., 2002), while ET-3 has 100-fold lower affinity for ET_A_R than ET_B_R (Horinouchi et al., 2013). Both endothelin receptors are desensitized through GPCR kinase type-2 (GRK-2) dependent mechanisms (Freedman et al., 1997), employing a pathway utilized in the desensitization of many GPCRs, including those that desensitize BK-mediated responses.

The nonapeptide BK belongs to the kinin family and is synthesized from inactive kininogen precursors following tissue injury (Dray and Perkins, 1993). Similar to ET-1, BK signals through two Gαq coupled receptors, BK type-1 and type-2 receptors (B_1_R and B_2_R, respectively; Gutowski et al., 1991; Marceau et al., 1998). BK signals predominantly through B_2_R, which is also the dominant isoform widely expressed in multiple tissue types under normal physiological conditions (Calixto et al., 2004). On the other hand, B_1_Rs are usually not expressed under physiological conditions but upregulated in response to inflammation and tissue injury (Calixto et al., 2004; Campos et al., 2006; Luiz et al., 2010). Furthermore, BK has significantly higher affinity for B_2_R over B_1_R (Calixto et al., 2000, 2004; Campos et al., 2006). Similar to ET receptors, B_2_R activation leads to receptor desensitization via GRK-2-dependent endocytosis (Blaukat et al., 1996; Haasemann et al., 1998). Therefore, we focused our studies with BK on the B_2_R system.

Both ET and BK receptors signal through Gαq/11 protein-coupled receptors that stimulate inositol 1,4,5-trisphosphate (IP_3_) and diacylglycerol (DAG) production via activation of phospholipase C (PLC; Takigawa et al., 1995; Nelson et al., 2003; Khodorova et al., 2009; Petho and Reeh, 2012). Although additional Gα-subunit signaling mechanisms have been linked to both BK (Gαi; Ewald et al., 1989 and Gαs; Liebmann et al., 1996) and ET (Gαi; Shraga-Levine and Sokolovsky, 2000 and Gαs; Jaureguiberry et al., 2004) receptors, Gαq/11 is identified as the dominating target of BK and ET receptor activation in peripheral neurons (Davenport, 2002; Leeb-Lundberg et al., 2005). For this reason, we focused our studies on calcium responses following activation of the classical PLC pathway downstream from Gαq/11, even though alternative pathways including protein kinase A, protein kinase C, and several mitogen-activated protein kinases are also activated by Gαq/11-coupled processes. IP_3_ binds to receptors expressed on endoplasmic reticulum (ER) to induce calcium release from intracellular stores. The subsequent decrease of Ca^2+^ concentration in intracellular stores activates store-operated calcium entry (SOCE) channels, further increasing intracellular calcium accumulation (Putney, 1986). Focused investigations have since revealed molecular elements, stromal interaction molecule 1 (STIM1) and Orai1 as important modulators of SOCE function, as STIM1 serves as an ER-localized Ca^2+^ sensor (Liou et al., 2005), and Orai1 act as pore-forming subunits (Prakriya et al., 2006). The second coiled-coil domain in the cytosolic C-terminus of STIM1 interacts with and activates Orai1 following prior activation of its Ca^2+^-sensing EF hand domain (Prakriya et al., 2006; Muik et al., 2012). As Ca^2+^concentration in the ER lumen falls below ∼300 µM, STIM1 oligomerizes, translocates, and interacts with Orai1 to activate SOCE channels (Cahalan, 2009). STIM1 also regulates the activity of other calcium channels, including the transient receptor potential canonical channel (TRPC) ion channel family, defining their function as SOC channels (Yuan et al., 2007). Interestingly, both BK and ET regulate TRPC channel activities; ET-1 regulates TRPC3, 5, 6, 7 (Horinouchi et al., 2011), whereas BK regulates TRPC1, 3, 4, and 6 (Liou et al., 2005; Huang et al., 2011; Suzuki et al., 2011). Taking under consideration the expression pattern of TRPC ion channels in both trigeminal ganglia (TG) and DRG sensory neurons (Alkhani et al., 2014), their function as SOCE channels and their existence as downstream targets from Gαq/11-signaling (importantly TRPC3; Peppiatt-Wildman et al., 2007), we investigated functional coupling between TRPC3, Orai1 channels and ET-1, and BK receptors in rat TG sensory neurons. However, spontaneous nociceptive behavioral measures were conducted on hindpaw tissues innervated by DRG afferent neurons to reduce output ambiguity that occurs in tissues innervated by TG afferent neurons (Krzyzanowska and Avendano, 2012). TG neurons were used as they provide more neurons/ganglia than those isolated from dorsal root ganglia tissue, allowing us to reduce the number of animals used for this study. Importantly, sensory neurons from both the trigeminal and dorsal root ganglia yield similar expression patterns for ET and BK receptors (Pomonis et al., 2001; Sugiura et al., 2002; Chichorro et al., 2009; Luiz et al., 2010; Yamamoto et al., 2013). Here, we identify divergence in calcium signals generated downstream from ET_A_R and B_2_R activation with respect to SOCE channels.

## Material and Methods

### Reagents

The following reagents were used: ET-1 (Sigma Aldrich, St. Louis, MO), BK (Sigma Aldrich, St. Louis, MO), PyR3 (Tocris Bioscience, Bristol, United Kingdom), GdCl_3_ (Tocris Bioscience, Bristol, United Kingdom), and U73122 (Tocris Bioscience, Bristol, United Kingdom).

### Animals

All procedures utilizing animals were approved by the Institutional Animal Care and Use Committee of the University of Texas Health Science Center at San Antonio and were conducted in accordance with policies for the ethical treatment of animals established by the National Institute for Health. Male Sprague Dawley rats, 175 to 200 g in weight (Charles River Laboratories, Wilmington, MA), were used for behavior analyses and TG dissection.

### Cell Culture

TG neurons were cultured as previously described (Jeske et al., 2006). In short, TG were bilaterally removed from male Sprague Dawley rats, placed in ice-cold Hanks’ balanced salt solution (HBBS) medium and washed twice with HBBS. TGs were dissociated by simultaneous collagenase and dispase treatment and gentle trituration after 25 and 45 min of incubation (37 ℃, room air). Cells were washed twice to remove the digestive enzymes and placed on poly-d-lysine/laminin-coated cover slips (Corning, Bedford, MA) in Dulbecco’s Modified Eagle Medium supplemented with 10% fetal bovine serum, 1% penicillin/streptomycin, 1% glutamine, 100 ng/ml nerve growth factor. Cultures were maintained at 37 ℃, 5% CO_2_, and grown for 1 to 2 days for calcium imaging experiments.

### Nucleofection

For single-cell PLC activity experiments, TG neurons were nucleofected with 2 µg of GFP-PLC∂-PH (a kind gift from Nikita Gamper University of Leeds, UK; Gamper et al., 2004) according to manufacturer recommendations (Lonza, Allendale, NJ) prior to cover slip placement. Experiments were performed 2 days after procedure.

### Measurements of Inositol Phosphate Accumulation

ET-1- and BK-stimulated inositol monophosphate (IP) accumulation in primary sensory neuronal cultures was measured as previously described (Rowan et al., 2014). Neuronal cultures grown in 96-well plates were labeled with 2 µCi/ml [^3^H]myoinositol for 24 hr before experiments. After labeling cells were washed three times with 500 µl HBSS that contained 20 mM 4-(2-hydroxyethyl)-1-piperazineethanesulfonic acid (HEPES) and were preincubated in HBSS containing 20 mM HEPES and 20 mM LiCl for 15 min at 37 ℃ in room air. Cells were incubated with various concentrations of ET-1 and BK (25 min, 37 ℃) and reactions were terminated by the addition of ice-cold formic acid. Total [^3^H]IPs were separated by ion exchange chromatography and measured by liquid scintillation spectrometry. Data are expressed as a percentage increase above basal IP accumulation.

### Calcium Imaging

To measure single-cell intracellular Ca^2+^ levels, the dye Fura-2 AM (2 µM; Molecular Probes, Carlsbad, CA) was incubated with cultured neurons for 30 min at 37 ℃ in the presence of 0.05% Pluronic (Calbiochem/EMD Biosciences, Gibbstown, NJ). Fluorescence was detected with a Nikon Eclipse Ti-U microscope fitted with a 20×/0.8 NA Fluor objective. Fluorescence images from 340 nm to 380 nm excitation wavelengths were collected and analyzed with MetaFluor Software (MetaMorph, Web Universal Imaging Corporation, Downingtown, PA). All Ca^2+^ measurements were performed at RT. To asses single-cell Ca^2+^responses after ET-1 or BK stimulation, cells were continuously perfused with standard extracellular solution (SES) that contained (in mM) 140 NaCl, 5 KCl, 2 MgCl_2_, 2 CaCl_2,_ 10 HEPES, and 10 D(+)-glucose. After 60 s baseline was established 100 nM of ET-1 or BK was applied and net changes in Ca^2+^ were calculated. In recordings where cells underwent double treatment with ET-1 and BK, neurons were washed for 5 min with SES. To assess SOCE stimulated by ET-1 or BK, cultured neurons were perfused with Ca^2+^ free buffer (140 mM NaCl, 5 mM KCl, 2 mM MgCl_2_, 0.5 EGTA, 10 mM D-(+)-glucose, 10 mM HEPES, pH 7.4) and after baseline was established, ET-1 (100 nM) or BK (100 nM) was administered for 30 s. Following 5 min washout, 2 mM Ca^2+^ was introduced to the extracellular environment. Ratiometric data were converted to [Ca^2+^]_i_ by using the equation [Ca^2+^]_i_ = *K*[(*R* − *R*_min_)/(*R*_max_ − *R*)]. Values of *K*, *R*_min_, and *R*_max_ are determined with use of calibration kit (Invitrogen, Oregon). Data collected with calibration kit allow generation of a standard curve, which can then be used to convert fura-2 fluorescence measurements obtained from experimental samples into estimates of free Ca^2+^ concentration. The plot of the log of [Ca^2+]^_free_ (x-axis) versus the log of {[(*R* − *R*_min_)/(*R*_max_ − *R*)]} yields a straight line, the x-intercept of which is the log of *K*. *R* is the ratio of 510 nm emission intensity with excitation at 340 nm, to 510 nm emission intensity with excitation at 380 nm; *R*_min_ is the ratio at zero free Ca^2+^; *R*_max_ is the ratio at saturating Ca^2+^.

### Animal Behavior

All injections were given intraplantarly in 25 µl volumes via 28-gauge needle inserted through the lateral footpad just under the skin to minimalize tissue damage. Drugs stocks were dissolved in phosphate buffered saline (PBS) or PBS 2% Tween20. After injection (Time 0), animals were freely moving, on a flat surface enclosed by Plexiglas cage. Nocifensive behavior was defined as hindpaw flinching, lifting, and licking. Animals were observed for 40 min and behavior was quantified every 5 min.

### Data Analysis

For detail statistical analysis, GraphPad Prism 4.0 software was used (Graphpad Software, Inc., San Diego, CA). Data were given as mean ± standard error of the mean (Haasemann et al., 1998), with the value of *n* referring to the number of analyzed cells per group. Concentration–response data for ET-1 - and BK-mediated IP accumulation were fit to a logistic equation using nonlinear regression analysis to provide estimates of maximal response (*E*_max_) and potency (EC_50_) using Prism software. The significant difference between groups was assessed by one-way and two-way analysis of variance (Pechanova and Simko, 2007), with Bonferroni post hoc correction as required. Two conditions were compared using paired or unpaired *t*-test. A difference was accepted as significant if *p* < .05, *p* < .01, or *p* < .001, marked *, **, and ***, respectively. NS = not significant.

## Results

### ET-1 and BK Cause Nocifensive Behavior in Rats

**Figure 1.**
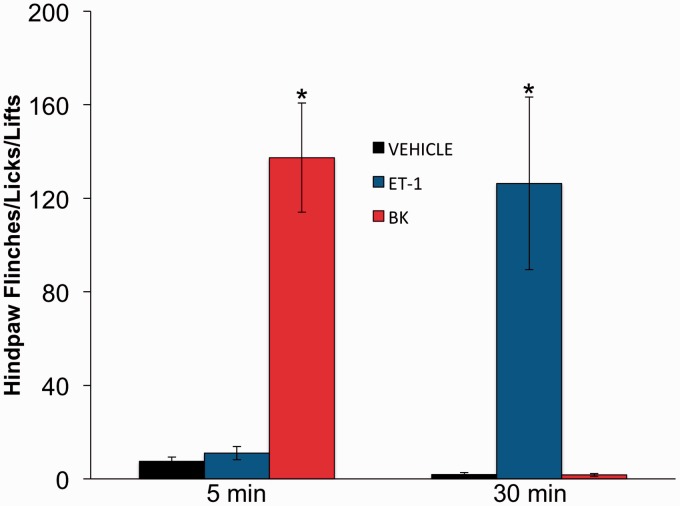
**Divergence in rat nocifensive responses following ET-1 and BK injection**. Nocifensive behavior observed after intraplantar injection of 25 µl ET-1 (10 nM), BK (10 nM), or PBS (vehicle) into rat hindpaw. 0 = *time of injection*. Number of hindpaw flinches, lifts, and licks were counted and reported at two time points after injection, 5 min and 30 min. Statistical significance was assessed by two-way ANOVA with Bonferroni correction, *n* = 6–9, *p < .05. **p < .01. ***p < .001. ET-1 = endothelin-1; BK = bradykinin; PBS = phosphate buffered saline; ANOVA = analysis of variance.

Inflammatory mediators induce nocifensive behaviors in multiple behavioral models (Gokin et al., 2001; Khodorova et al., 2002). Here, we aimed to directly compare the time frames of nocifensive behavior (flinching, licking, and lifting of injected paw) following intraplantar injection of rats with ET-1 (10 nM in 25 µl), BK (10 nM in 25 µl), or vehicle (PBS, 25 µl). As illustrated in Figure 1, statistical comparisons of blinded observations following intraplantar injection of ET-1 or BK into the hindpaw revealed significant differences at differential time points. Following BK administration, rat nocifensive behavior peaked 5 min after injection, while ET-1 injection produced similar nocifensive behavior, but not until 30-min postinjection. As both of these inflammatory mediators signal through similar Gαq-coupled mechanisms, our study progressed along the signaling pathway to investigate sensory neuronal Ca^2+^ accumulation.

### Single-Cell Ca^2+^Responses Triggered by ET-1 and BK in TG Neurons

**Figure 2.**
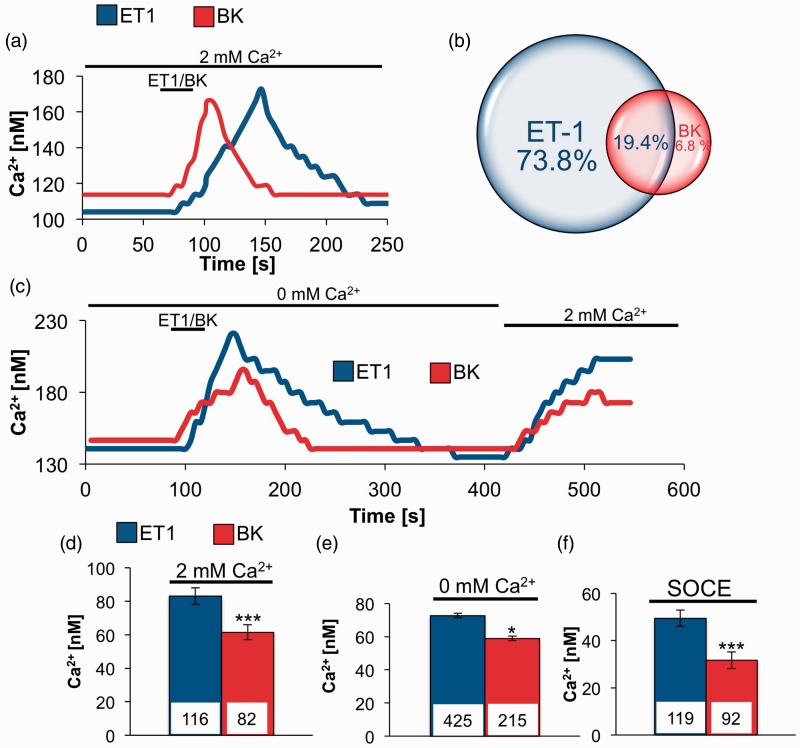
**Single cells Ca^2+^ responses in TG neurons induced by ET-1 and BK treatment. (**a) Original tracings from single-cell Ca^2+^ measurements showing changes in intracellular Ca^2+^concentration due to ET-1 or BK stimulation in 2 mM Ca^2+^ extracellular environment. (b) Graphic representation of frequency of responses to ET-1 and BK treatment in same set of TG neurons. After establishing baseline cells were treated with 100 nM ET-1, after 5 min recovery time, 100 nM BK was administered. Data are expressed as percentage of all responding cells (*n =* 103). (c) Original tracings from single-cell Ca^2+^ measurements in rats TGs with design to activate SOC channels. After establishing baseline in 0 mM Ca^2+^ solution, cells were treated for 30 s with ET-1 (100 nM) or BK (100 nM) followed by 5 min washout and addition of 2 mM Ca^2+^ to extracellular environment. (d) Summary of increase in cytosolic Ca^2+^concentration induced by ET-1 (100 nM) or BK (100 nM) in 2 mM of extracellular Ca^2+^. (e) Summary of increased intracellular Ca^2+^ concentration due to release from intracellular stores after administration of ET-1 or BK in 0 mM Ca^2+^ extracellular environment. (f) Summary of SOCE in rat TGs activated by depletion of intracellular stores following administration of ET-1 or BK. Statistical significance was assessed by unpaired *t-*test, *p < .05. **p < .01. ***p < .001. TG = trigeminal ganglia; ET-1 = endothelin-1; BK = bradykinin; SOC = store-operated Ca^2+^; SOCE = store-operated Ca^2+^ entry; TG = trigeminal ganglia.

We next decided to characterize single-cell Ca^2+^ responses triggered by ET-1 and BK by real-time Ca^2+^ imaging, starting with physiological extracellular solution containing 2 mM Ca^2+^ (Figure 2(a) and (d)) Under those conditions, ET-1 treatment effected in significantly higher increase in intracellular Ca^2+^ concentration when compared with BK (Figure 2(d)). In following set of measurements, we used protocol that was designed to isolate Ca^2+^ entry due to SOC channels activity. After baseline in Ca^2+^ free solution was established, cells were treated for 30 s with ET-1 (100 nM) or BK (100 nM) and changes in intracellular calcium concentration were analyzed. ET-1 triggered significantly higher calcium release from intracellular stores when compared with BK-induced responses (Figure 2(c) and (e)). After cells had time to recover from ET-1 or BK stimulation, we exchanged extracellular solution from Ca^2+^ free solution to one containing 2 mM Ca^2+^, observed intracellular Ca^2+^ increase was due to activity of SOC channels. Data summarized in Figure 2(f) showed that ET-1-induced Ca^2+^ depletion caused significantly higher SOC channels activation than that caused by BK. We were also interested in the frequency of coexpression of ET_A_R and B_2_R in TG neurons. To that end, after we established the baseline, we treated cells with ET-1 and after 5 min of recovery, the same cells were treated with BK and the frequency of events is illustrated in Figure 2(b); 20.8% of cells that responded to ET-1 also responded to BK stimulation. On the other hand, 74.1% of cells that showed sensitivity to BK treatment also showed response to ET-1.

### ET-1 and BK Activation of PLC in Population and Single-Cell Measurements

**Figure 3.**
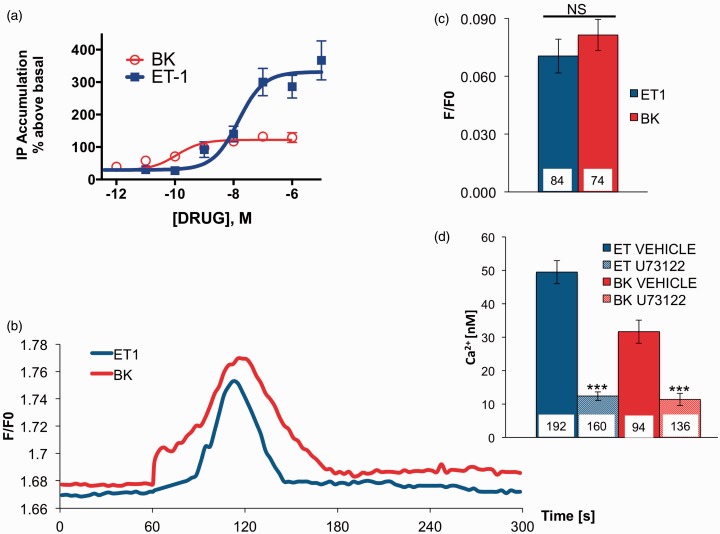
**ET-1- and BK-induced activation of PLC in population and single-cell measurements. A.** TG cultures were incubated for 30 min (37 ℃) with the indicated concentration of ET-1 or BK. Total accumulation of IP was determined as described in Methods section. Data shown are the mean percentage above basal IP accumulation ± SEM, *n* = 6. (b) TG neurons nucleofected with GFP-PLC∂-PH and underwent ET-1 (100 nM) or BK (100 nM) stimulation. Translocation of membrane-localized PLC∂-PH to the cytosol reports PIP_2_ hydrolysis by ET-1 (100 nM) or BK (100 nM) stimulation. Normalized (to the background values) cytosolic GFP fluorescence excited at 470 nm during ET-1 and BK stimulation is displayed. (c) Summary of net change in GFP cytosolic fluorescence during ET-1 and BK application. (d) Summary of Ca^2+^measurements of store-operated Ca^2+^ entry in TGs. Ca^2+^ entry was induced by store depletion by stimulation of cells with ET-1 (100 nM) or BK (100 nM) in the presence or absence of U73122 (10 µM, PLC inhibitor). Statistical significance was assessed by unpaired *t*-test, or two-way ANOVA with Bonferroni post hoc correction, *p < .05. **p < .01. ***p < .001. ET-1 = endothelin-1; BK = bradykinin; PLC = phospholipase C; IP = inositol monophosphate; SEM = standard error of the mean; ANOVA = analysis of variance; PIP_2_ = phosphatidylinositol 4,5-bisphosphate; GFP = green fluorescent protein.

To examine possible mechanisms that would explain the variability in calcium accumulation following ET-1 and BK treatment of sensory neurons, we focused on PLC activation, as it is the first molecular reaction that takes place after activation of ET_A_R and B_2_R. IP is one metabolite that accumulates in the presence of LiCl after hydrolysis of phosphatidylinositol 4,5-bisphosphate (PIP_2_) and is broadly used as an index of PLC enzymatic activity (Rowan et al., 2014). When IP accumulation was measured in sensory neuron cultures after treatment with ET-1 or BK, significant differences between maximal responses of the agonists were observed (Figure 3(a)). However, given that sensory neuron cultures are heterogeneous and significant differences observed could be due to high expression of ET_A_R in non-neuronal cells, we analyzed PLC activity on a single-cell level (Gamper et al., 2004). To this end, we nucleofected cultured sensory neurons with green fluorescent protein (GFP)-tagged PLC∂ pleckstrin homology domain cDNA, which translocates to the cytosol from the plasma membrane following activation of PLC and subsequent PIP_2_ hydrolysis. When PLC activation was compared in cells stimulated with ET-1 or BK, no significant differences were observed (Figure 3(b) to (c)), suggesting that on a single-cell level, ET-1 and BK activate PLC similarly. To further investigate a potential role for PLC in sensory neuronal SOCE, we measured real-time calcium accumulation in the presence of U73122 (10 µM, PLC inhibitor, Figure 3(d)). Under this condition, neither ET-1 nor BK was able to generate notable SOCE, indicating that activation of PLC is necessary for ET-1- and BK-mediated SOCE in cultured sensory neurons.

### Ion Channels That Contribute to SOCE in TG Neurons

**Figure 4.**
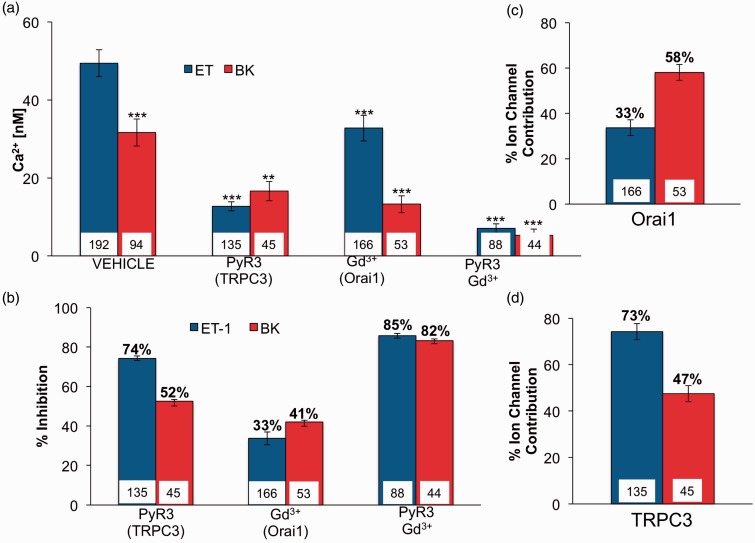
**Pharmacological characterization of ET-1 and BK activated SOCE in rat TG neurons.** (a) Summary of changes in single-cell intracellular Ca^2+^ concentration as a measurement of SOCE induced by administration of ET-1 or BK in the presence or absence of various ion channel inhibitors: PyR3 (10 µM, TRPC3), Gd^3+^ (10 µM, ORAI), or combination of both. (b) Data from panel (a) as represented by percentage of maximal response inhibited by PyR3, Gd^3+^, or combination of both. (c) Summary of Orai1 ion channel contribution to ET-1- and BK-induced SOCE in rat TG neurons (single-cell measurements). (d) Summary of TRPC3 ion channel contribution to ET-1- and BK-induced SOCE in rat TG neurons (single-cell measurements). Statistical significance was assessed by two-way ANOVA with Bonferroni correction, *n* = 6–9, *p < .05. **p < .01. ***p < .001. ET-1 = endothelin-1; BK = bradykinin; SOCE = store-operated Ca^2+^ entry; TG = trigeminal ganglia; TRPC3 = transient receptor potential canonical channel 3; ORAI = ▪ ▪ ▪; ANOVA = analysis of variance.

Previous studies indicate similar expression patterns of ET_A_R and B_2_R in primary nociceptors (Gemes et al., 2011; Alkhani et al., 2014) and that both ET-1 and BK activate SOCE, due to PLC-dependent IP_3_ production following receptor activation (Figure 2, Putney, 2011; Tykocki et al., 2014). Additionally, previous reports recognize roles for Orai1 and TRPC3 as SOCE channels in sensory neurons (Gemes et al., 2011; Alkhani et al., 2014). Therefore, we next investigated Orai1 and TRPC3 as possible candidates that contribute to ET-1- or BK- induced SOCE activation in TG neurons. Pharmacological SOCE antagonists were used to determine single-cell calcium response sensitivities to ET-1 and BK treatment (Figure 4). SOCE triggered by ET-1 was decreased by 74% when PyR3 (TRPC3 blocker) was present (Figure 4(a) and (b)), whereas, Gd^3+^ (Orai1 blocker) caused a 33% decrease in calcium response (Figure 4(a) and (b)). In contrast, when BK was the agent that activated SOCE in TG neurons, PyR3 treatment resulted in 52% reduction in SOCE response (Figure 4(a) and (b)); moreover, Gd^3+^ inhibited calcium response by 41% (Figure 4(a) and (b)). When we translate these data to represent specific ion channel contribution to ET or BK response, TRPC3 contributes 73% to ET-1- and 47% to BK-activated SOCE activities and, Orai1 contributes to 33% of ET-1- and 53% of BK-triggered SOCE in sensory neurons (Figure 4(c) and (d)).

### Inhibition of Orai1- and TRPC3-Modified Nocifensive Behavior in Rats

**Figure 5.**
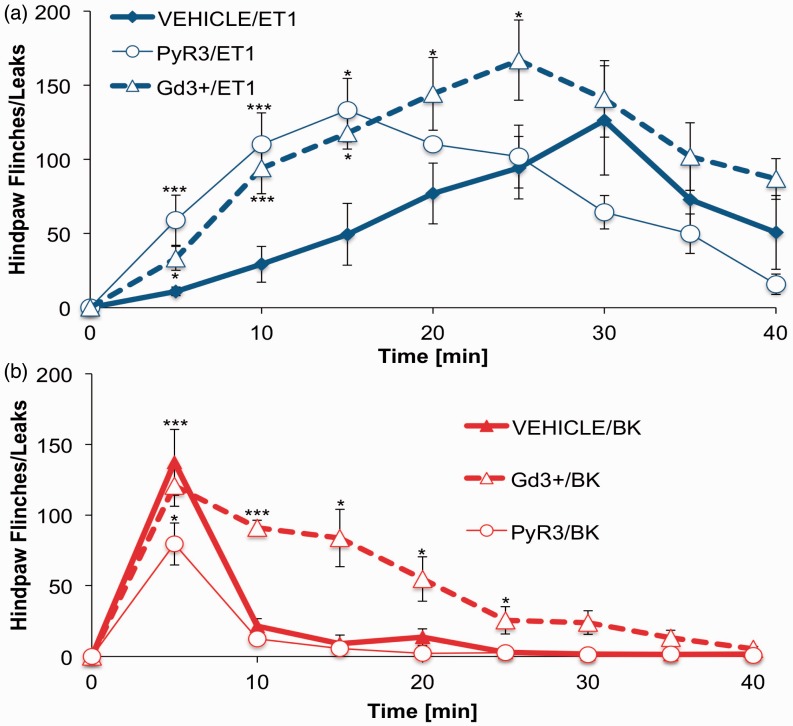
**Nocifensive behavior in rats after administration of ET-1 or BK.** Two intraplantar injections were administered in 5 min intervals. First injection consisted of 25 µl of PyR3 (100 nM), Gd^3+^ (100 nM), or PBS as vehicle. Second contained 25 µl ET-1 (10 nM) and BK (10 nM). Nocifensive behavior was recorded throughout the time course of experiment (40 min) and quantified every 5 min. (a) Summary of ET-1-induced behavior in rats in the presence or absence of ion channel inhibitor injected 5 min prior to peptide injection. (b) Summary of BK-induced behavior in rats in the presence or absence of ion channel inhibitor, injected 5 min prior to peptide injection. Statistical significance was assessed by two-way ANOVA with Bonferroni correction, *n* = 6–9, *p < .05. **p < .01. ***p < .001. ET-1 = endothelin-1; BK = bradykinin; PBS = phosphate buffered saline; ANOVA = analysis of variance.

As described earlier, ET-1 and BK treatment induces significant nocifensive behavior in rats (Figure 1). Following *in vitro* experiments that identify two ion channels that contribute to ET-1- and BK-mediated responses, nocifensive behavior experiments were repeated with specific ion channel inhibitors, PyR3 was used as a TRPC3-specific inhibitor (Alkhani et al., 2014) and Gd^3+^ was injected to block Orai1 activity (Gemes et al., 2011). Intraplantar injection of 100 nM (25 µl) inhibitor (PyR3 or Gd^3+^) into the hindpaw was followed by 10 nM (25 µl) injection of ET-1 or BK 5 min later. Inhibition of Orai1 with Gd^3+^ resulted in a significant increase in ET-1-triggered nocifensive behavior through the first 25 min of observation (Figure 5). PyR3 inhibition of TRPC3 prior to ET-1 administration resulted in a leftward shift in the time course of response, inducing an earlier onset of nociceptive responses; however, the magnitude of response was unchanged. As for BK-induced nocifensive behavior, Orai1 inhibition did not change the maximal response; however, the recovery time was prolonged when compared with BK alone. Furthermore, TRPC3 inhibition by PyR3 significantly decreased peak BK nocifensive behavior at the 5-min time point, suggesting that activation of TRPC3 contributes to BK-mediated nocifensive responses.

## Discussion

Sensory neurons, especially slow conducting C-type fibers, can remain in quiescent states for long periods of time (Schmidt et al., 1995). During that time, inactivity of voltage gated Ca^2+^ channels and continuous membrane Ca^2+^ATPase activity could deplete intracellular Ca^2+^ stores. However compensatory SOCE mechanisms prevent store depletion and support cellular viability (Wanaverbecq et al., 2003; Gemes et al., 2011). Very few published studies have addressed the role(s) of SOCE in sensory neurons. These earlier works identify that extracellular Ca^2+^ is required to maintain calcium homeostasis in resting neurons, as they show decreased Ca^2+^ levels within minutes after placement in Ca^2+^-free solution (Usachev and Thayer, 1999; Gemes et al., 2011). Additionally, when quiescent neurons recover in the presence of 2 mM extracellular Ca^2+^, stores are restored within 10 min (which does not occur in a Ca^2+^-free environment), demonstrating neuronal dependence on SOCE for replenishing Ca^2+^ stores after a release event (Gemes et al., 2011). Furthermore, negative manipulations of SOCE can deplete intracellular stores, contributing to pathological conditions following neuropathy (Rigaud et al., 2009). However, inflammatory GPCR activation can also cause store depletion following acute injury, although mediators that activate the same receptor signaling mechanisms often yield differential cellular results.

In the present study, we investigated the differential sensory neuron activation profiles for the inflammatory mediators ET-1 and BK. Both mediators signal through Gαq/11 protein-coupled pathways, activate PLC, stimulate increased intracellular Ca^2+^ accumulation, and undergo rapid GRK2-dependent desensitization (Khodorova et al., 2009; Petho and Reeh, 2012; Blaukat et al., 1996; Freedman et al., 1997; Haasemann et al., 1998). However, we report differential nociceptive behavior patterns and calcium accumulation indices following either ET-1 or BK administration. To address this, we examined SOCE channels farther downstream from receptor activation and discovered a unique pathway association between ET-1 and TRPC3 separate from a more balanced pathway linking B_2_R activation to both TRPC3 and Orai1. This differential pathway association is especially unique, given the similarities between the receptors activated and their immediate downstream targets.

In many cell types, BK stimulates transient increases in intracellular Ca^2+^, only to return to baseline within minutes following treatment (Pinheiro et al., 2013). In contrast, ET-1 stimulates transient increases in free Ca^2+^concentration in aortic smooth muscle, and elevated Ca^2+^ levels can be maintained for up to 60 min (Miwa et al., 1999). Results presented here demonstrate clear parallels between nocifensive behavior time course and calcium accumulation. BK induced a short-lived Ca^2+^signal that translates into quickly resolved nocifensive behavior (Figure 1). In contrast, ET-1 fostered a prolonged Ca^2+^elevation that contributes to extended nocifensive behavior (Figure 1). These observations, combined with previous knowledge about signal transduction pathway downstream of ET_A_R and B_2_R, suggest that second messenger pathway(s) bifurcate to contribute to divergent responses. At the beginning of our investigation, we established that in single-cell measurements, both ET-1 and BK stimulation activate PLC to the same degree (Figure 3(b) and (c)) and mobilize Ca^2+^ from intracellular stores (Figure 2(c) and (e)). However, a consequence of store depletion is the activation of various SOCE channels. Therefore, we investigated SOCE channels activated by ET-1 and BK to clarify observed discrepancies.

Orai1 and TRPC3 are among many SOCE channels that assist in intracellular Ca^2+^ homeostasis, and both are expressed and have been characterized in sensory neurons (Gemes et al., 2011; Alkhani et al., 2014). Commercially available pharmacological antagonists of these channels were used to determine the roles of each in mediating the differential effects of ET-1 and BK. Results presented here demonstrate that ET-1 predominantly activates TRPC3, which contributes to 73% of ET-1-induced SOCE (Figure 4(d)). In contrast, BK-triggered SOCE is significantly more balanced between Orai1 and TRPC3, with slight preference toward Orai1 involvement (58%, Figure 4(c)). This variability in ion channel activation resulted in significant differences in the length and size of the Ca^2+^ signal. Importantly, this divergence in SOCE activation profiles could contribute to the differential nocifensive behaviors caused by ET-1 and BK. To further examine the roles of Orai1 and TRPC3 in ET-1 - and BK-induced behavior, we employed the same pharmacological inhibitors in our nocifensive behavior model.

SOCE antagonist results establish parallels between the functional coupling of Orai1 and TRPC3 channels in single-cell responses and nocifensive behavior. Orai1 suppression of neuronal excitability would explain significantly prolonged recovery time after BK injection in rats that were pretreated with the Orai1 blocker, Gd^3+^ (Figure 5(b)). Furthermore, PyR3 inhibition of TRPC3 significantly reduced BK-induced peak nocifensive behavior. This finding agrees with the robust BK-induced inflammatory response observed by ourselves (Figure 1) and others (Petho and Reeh, 2012). However, neither of the SOCE antagonists reduced peak ET-1 nocifensive behavior, and in both instances, affected the time-to-peak response ratio. Both PyR3 inhibition of TRPC3 and Gd^3+^ inhibition of Orai1 reduced the time required for ET-1 administration to reach peak nocifensive behavior. These results may simply be due to the quick behavioral response of BK versus the slower response time for ET-1or may involve additional underlying factors.

It has been reported that TRPC3 plays vital role in DRG neurons sensitization triggered by exposure to inflammatory mediators (Alkhani et al., 2014). This may explain why the ET-1 signal, which is very strongly biased toward TRPC3 activation, resulted in prolonged nocifensive responses. Also, TRPC3 inhibition would alter this behavior through decreased sensitization, resulting in reduced nocifensive action. Further modification of rat behavior was observed when Orai1 was inhibited prior to ET-1 injection. Interestingly, Orai1 activity has been shown to suppress neuronal excitability, while inhibition increases neuronal burst firing (Gemes et al., 2011). Here, this mechanism would explain the increased nocifensive responses in animals pretreated with the Orai1 inhibitor Gd^3+^ prior to ET-1 administration (Figure 5(a)). Indeed, as ET_A_R downstream signaling targets Orai1 to a smaller degree than TRPC3, the shift in nocifensive behavior response time shift could be affected. Importantly, TRPC3 inhibition produced greater changes in nocifensive behavior, similar to single-cell studies. Despite the lack of mirrored results between single-cell studies and nociceptive behavior, important findings appear to indicate divergent signaling mechanisms that contribute to our initial behavioral findings.

**Figure 6.**
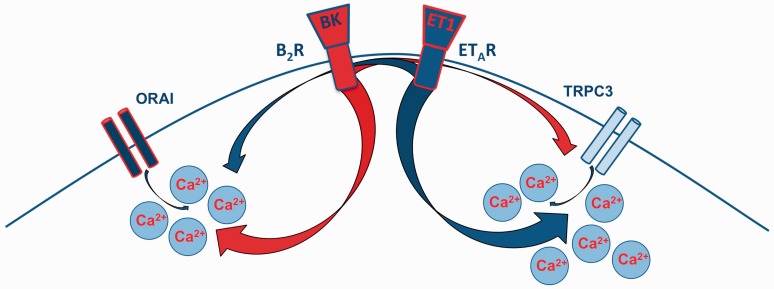
**The graphic representation of ET-1 and BK signaling with emphasis on prevalent ion channels activated downstream from ET_A_R and B_2_R.** ET-1 = endothelin-1; BK = bradykinin; ET_A_R = endothelin-A.

Taken together (Figure 6), TRPC3 is the primary target of ET-1 signaling in TG sensory neurons and its activity contributes to prolonged elevation of intracellular free Ca^2+^ levels and the perpetuation of nocifensive responses. In contrast, BK triggers short-lived transient increases in intracellular Ca^2+^ through dominant activation of Orai1, contributing to a rapid, but abbreviated onset of nocifensive behavior. Further studies into this divergent signaling behavior could prove important to alleviating acute hyperalgesia.

## Summary

Endothelin and bradykinin are inflammatory mediators that bind to receptors on sensory neurons to stimulate various molecular pathways. In this investigation, we identified store-operated ion channels Orai1 and TRPC3 contribute to endothelin/bradykinin effects *in vitro* and *in vivo*.
